# Ronin overexpression induces cerebellar degeneration in a mouse model of ataxia

**DOI:** 10.1242/dmm.044834

**Published:** 2021-06-24

**Authors:** Thomas P. Zwaka, Marta Skowronska, Ronald Richman, Marion Dejosez

**Affiliations:** 1 Department for Cell, Regenerative and Developmental Biology, Icahn School of Medicine at Mount Sinai, New York, NY 10029, USA; 2 Black Family Stem Cell Institute, Icahn School of Medicine at Mount Sinai, New York, NY 10029, USA; 3 Huffington Center for Cell-Based Research in Parkinson's Disease, Icahn School of Medicine at Mount Sinai, New York, NY 10029, USA; 4 Department of Molecular and Human Genetics, Baylor College of Medicine, Houston, TX 77030, USA; 5 Jan and Dan Duncan Neurological Research Institute at Texas Children's Hospital, Houston, TX 77030, USA; 6 Howard Hughes Medical Institute, Baylor College of Medicine, Houston, TX 77030, USA

**Keywords:** Ataxia, Polyglutamine disease, Purkinje cells, Embryonic stem cells, Copy number variation

## Abstract

Spinocerebellar ataxias (SCAs) are a group of genetically heterogeneous inherited neurodegenerative disorders characterized by progressive ataxia and cerebellar degeneration. Here, we used a mouse model to test a possible connection between SCA and Ronin (Thap11), a polyglutamine-containing transcriptional regulator encoded in a region of human chromosome 16q22.1 that has been genetically linked to SCA type 4. We report that transgenic expression of Ronin in mouse cerebellar Purkinje cells leads to detrimental loss of these cells and the development of severe ataxia as early as 10 weeks after birth. Mechanistically, we find that several SCA-causing genes harbor Ronin DNA-binding motifs and are transcriptionally deregulated in transgenic animals. In addition, ectopic expression of Ronin in embryonic stem cells significantly increases the protein level of Ataxin-1, the protein encoded by Atxn1, alterations of which cause SCA type 1. This increase is also seen in the cerebellum of transgenic animals, although the latter was not statistically significant. Hence, our data provide evidence for a link between Ronin and SCAs, and suggest that Ronin may be involved in the development of other neurodegenerative diseases.

## Abstract (precis)

**Summary:** Ronin is a polyglutamine protein encoded in a region of human chromosome 16q22.1 linked to spinocerebellar ataxia type 4. Overexpression of Ronin in mouse cerebellar Purkinje cells leads to their loss and ataxia.

## INTRODUCTION

**Fig. 1.**
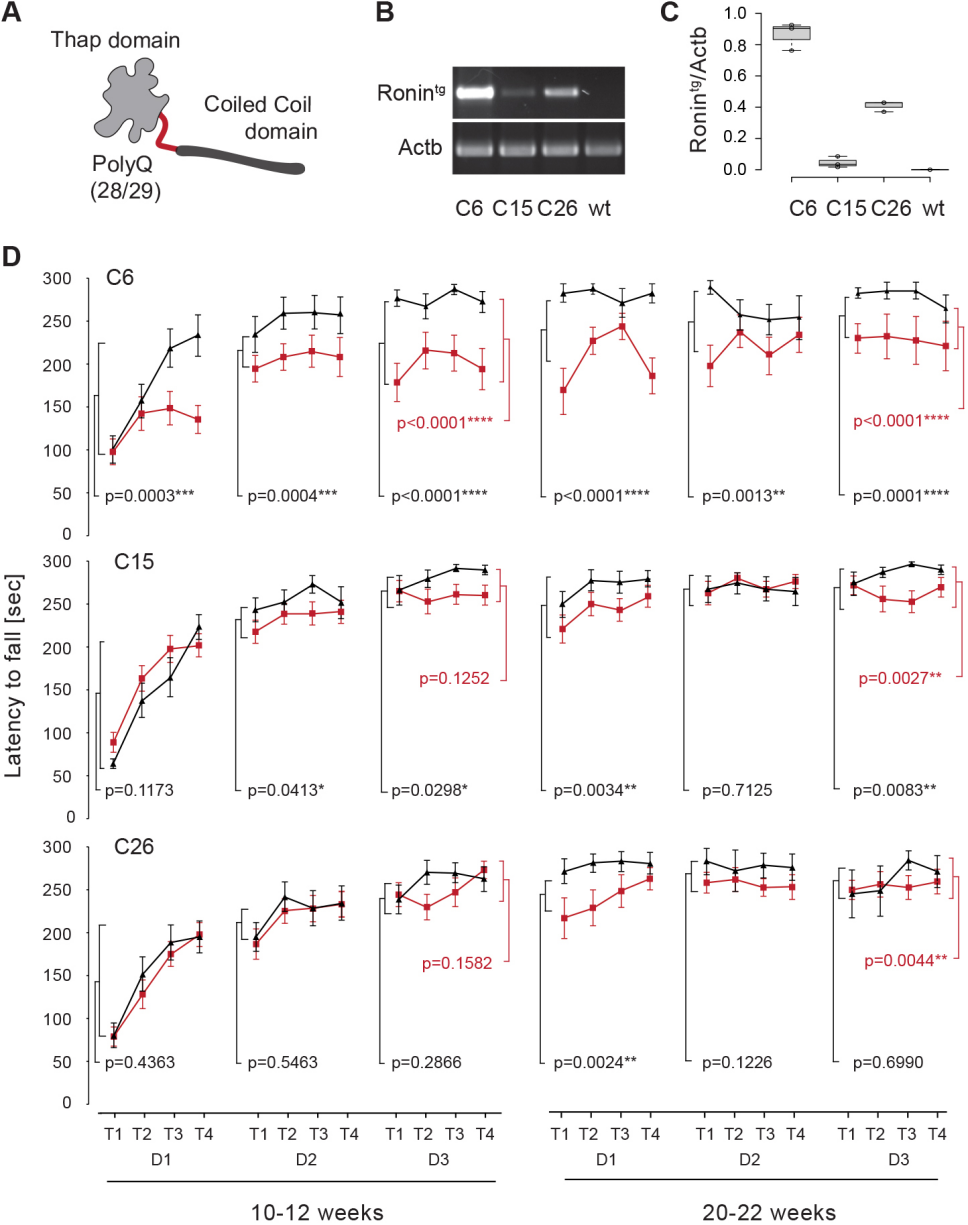
**Purkinje cell-specific transgenic expression of Ronin leads to reduced rotarod performance.** (A) Schematic of a Ronin monomer, in which the C-terminal Thap domain is separated from the N-terminal coiled coil domain by a 29/30 Q long polyQ tract. (B) RT-PCR analysis of mRNA isolated from cerebella of transgenic and wild-type (wt) animals from three independent mouse lines (C6, C15 and C26). (C) Quantification of the mRNA levels shown in B. Data are shown as box plots; each circle represents the signal from one animal. *n*=3 per line. The boxes represent the 25-75th percentiles, and the whiskers extend 1.5× the interquartile range from the 25th and 75th percentiles. The median is indicated. The center lines show the median. Actb, Actin B. (D) Rotarod analyses of a representative set of animals from transgenic L7-Ronin (Ronin^tg^) animals (red lines) from three different mouse lines (C6, C15 and C26) at 10-12 weeks (left) and 20-22 weeks (right) of age compared to wild-type littermates (black lines). The latency to fall in seconds in four trials (T1-T4) on three consecutive days (D1-D3) is shown. Data are mean±s.e.m. *P*-values were calculated using mixed hierarchical models and describe the difference between the performance average (four trials per animal) of wild-type control and Ronin^tg^ animals for each day of the trial (indicated in black) or all days (average of 12 trials per animal) combined (indicated in red). *n*=13, 24 or 21 for transgenic, and *n*=14, 13 or 7 for wild-type animals of lines C6, C15 and C26, respectively. *P≤0.05, **P≤0.01, ***P≤0.001, ****P≤0.0001.

Spinocerebellar ataxias (SCAs) are a genetically heterogeneous group of neurodegenerative diseases characterized by loss of motor coordination and balance. Despite the genetic heterogeneity, many ataxias show striking similarity and it is often difficult to distinguish between different types of SCAs based solely on clinical and pathological observations. The most common neuropathological feature of SCAs is the atrophy of the cerebellum that is accompanied by a characteristic loss of Purkinje cells. SCA type 1 (SCA1), at least five additional SCAs (SCA2, 3, 6, 7 and 17) and other neurodegenerative disorders, such as Huntington disease (HD), are caused by proteins with abnormally long polyglutamine tracts as common cause in pathogenesis (Durr, 2010; Schöls et al., 2004; Paulson, 2009; Orr and Zoghbi, 2007). Although the origin of many SCAs is still uncertain, there is an interesting polyglutamine repeat-encoding gene, *Ronin* (herein referring to *Thap11*), located in the genomic region on chromosome 16q22.1 (chr16: 66,095,251 and 74,034,030; hg38) that has been mapped to SCA type 4 (SCA4) disease (Flanigan et al., 1996; Hellenbroich et al., 2003, 2006). Ronin, a transcriptional regulator that is essential for embryonic development (Dejosez et al., 2008; Fujita et al., 2017), seems worth considering in the context of ataxias for a number of reasons. First, Ronin contains an average of 28/29 glutamine residues (ranging from 21-41) (Pandey et al., 2004), and thus, like Atxn1 (Suh et al., 2019), has one of the longest polyglutamine-coding stretches in the human genome (Butland et al., 2007; Fig. 1A; Fig. S1A). Second, Hcf1 (herein referring to Hcfc1), which is a binding partner of Ronin (Dejosez et al., 2008, 2010), was found to be part of the ataxia protein-protein interaction network (Lim et al., 2006; Zhang et al., 2020), and has been broadly implicated in neurodevelopmental disorders, including non-syndromic X-linked mental retardation (Ferrari et al., 2016; Huang et al., 2012; Vrabec et al., 2008; Turner et al., 2015; Jolly et al., 2015). Finally, mutations in Thap1, the highly similar gene cousin of Ronin, causes DYT6 dystonia (Fuchs et al., 2009; Mazars et al., 2010), which is an inherited movement disorder that affects basal ganglia and the cerebellum (Neychev et al., 2011; Prudente et al., 2014; Nibbeling et al., 2017; Ruiz et al., 2015).

Although evidence linking Ronin to SCA4 has been acknowledged (Pandey et al., 2004), sequencing efforts have failed to reveal any suspicious mutation or polyglutamine-coding repeat expansion in the SCA4-linked genomic region on chromosome 16q22.1 (Hellenbroich et al., 2005; Edener et al., 2011), and no abnormal polyglutamine aggregates were found in SCA4 patients (Hellenbroich et al., 2006). Hence, we argued, if Ronin were to be involved in the development of SCA4 or any other SCA, it would most likely entail alternative genetic causes. We hypothesized that the genetic alteration might involve aberrations, such as an increased gene dosage (copy number variation, CNV) or non-coding changes that alter mRNA stability or modulate the expression of its encoding gene. To test this idea, we investigated whether ectopic expression of Ronin in Purkinje cells can induce ataxia in mice.

## RESULTS

### Ronin is present in Purkinje cells throughout the cytoplasm and nucleus

SCAs are caused, at least in part, by cerebellar Purkinje cell degeneration. Hence, we first investigated whether Ronin protein is present in Purkinje cells. *In situ* hybridization data (Allen Mouse Brain Atlas, Lein et al., 2007) and our previous expression analysis in Ronin-lacZ reporter mice (Dejosez et al., 2008) already suggested that *Ronin* is expressed in this cell type at the RNA level. In line with this finding, immunostaining of sagittal cerebellar sections confirmed that Ronin protein is expressed in Purkinje cells. We found predominantly nuclear staining of Ronin in Purkinje cells of 5-week-old animals that weakened at 11 weeks and was not detectable at 58 weeks (Fig. S1B).

### Purkinje cell-specific transgenic expression of Ronin causes ataxia

The polyglutamine encoding CAG repeats of Ronin are interrupted by CAAs that guard against replication-dependent slippage expansions (Fig. S1C). As such, no expansions of the Ronin polyglutamine tract have been identified thus far and, furthermore, no other pQ unrelated mutations have been found in Ronin or elsewhere in the SCA4-linked genomic region on chromosome 16q22.1 (Hellenbroich et al., 2005; Edener et al., 2011). An alternative cause that has not been considered yet is increased gene dosage (e.g. CNVs) of Ronin. Therefore, we sought to test whether transgenic expression of normal Ronin protein might be sufficient to cause ataxia in mice. To accomplish this goal, we used a Pcp2/L7-Ronin-Flag construct (Fig. S2A; Smeyne et al., 1995) to overexpress transgenic *Ronin* (Ronin^tg^) specifically in Purkinje cells. Southern blot analysis (Fig. S2B) and RT-PCR (Fig. 1B,C) confirmed transgene integration and expression in second-generation animals at 5 weeks of age in three independent mouse lines (derived from founder animals C6, C15 and C26, respectively). The highest *Ronin* transgene (Ronin^tg^) levels were seen in line C6, followed by C26 and C15 (Fig. 1B,C). Inspection of the cerebellar morphology of the transgenic founder animals at 36 weeks of age (Fig. S2C) revealed a significant reduction in the cerebellar size in animal C6, and calbindin staining showed a severe loss of nearly all Purkinje cells (Fig. S2D). The cerebellum of animal C26 had developed a soft sponge-like structure (that disappeared in subsequent generations) and regional Purkinje cell loss, whereas the cerebellum of animal C15 did not show any gross morphological changes (Fig. S2C,D).

We next determined whether the Purkinje cell-specific expression of transgenic *Ronin* can induce ataxia by testing the rotarod performance of our transgenic mouse lines. The latency to fall was measured over the course of 5 min on an accelerating rotarod (Watase et al., 2002) at 10-12 weeks and 20-22 weeks of age. In these tests, transgenic animals of all three L7-Ronin lines showed significantly decreased performance compared to wild-type animals (Fig. 1D). The performance at 20-22 weeks matched the one on the last day of the previous round (10-12 weeks), indicating that they exhibited a training effect. Thus, the inability to stay on the rotarod did not appear to be caused by a general learning defect. Because transgenic animals from all three lines showed decreased rotarod performance, we find it unlikely that the transgene integration site itself influenced the observed phenotype. Furthermore, the severity of the phenotype correlated with the mRNA expression level of transgenic Ronin. Immunofluorescence staining of cerebellar sections confirmed the differences in Ronin expression between lines C6 (highest Ronin^tg^ RNA expression) and C15 (lowest Ronin^tg^ RNA expression) at the protein level, and revealed that the exogenous Ronin protein was primarily located in the Purkinje cell nucleus (Fig. S3A,B).

**Fig. 2.**
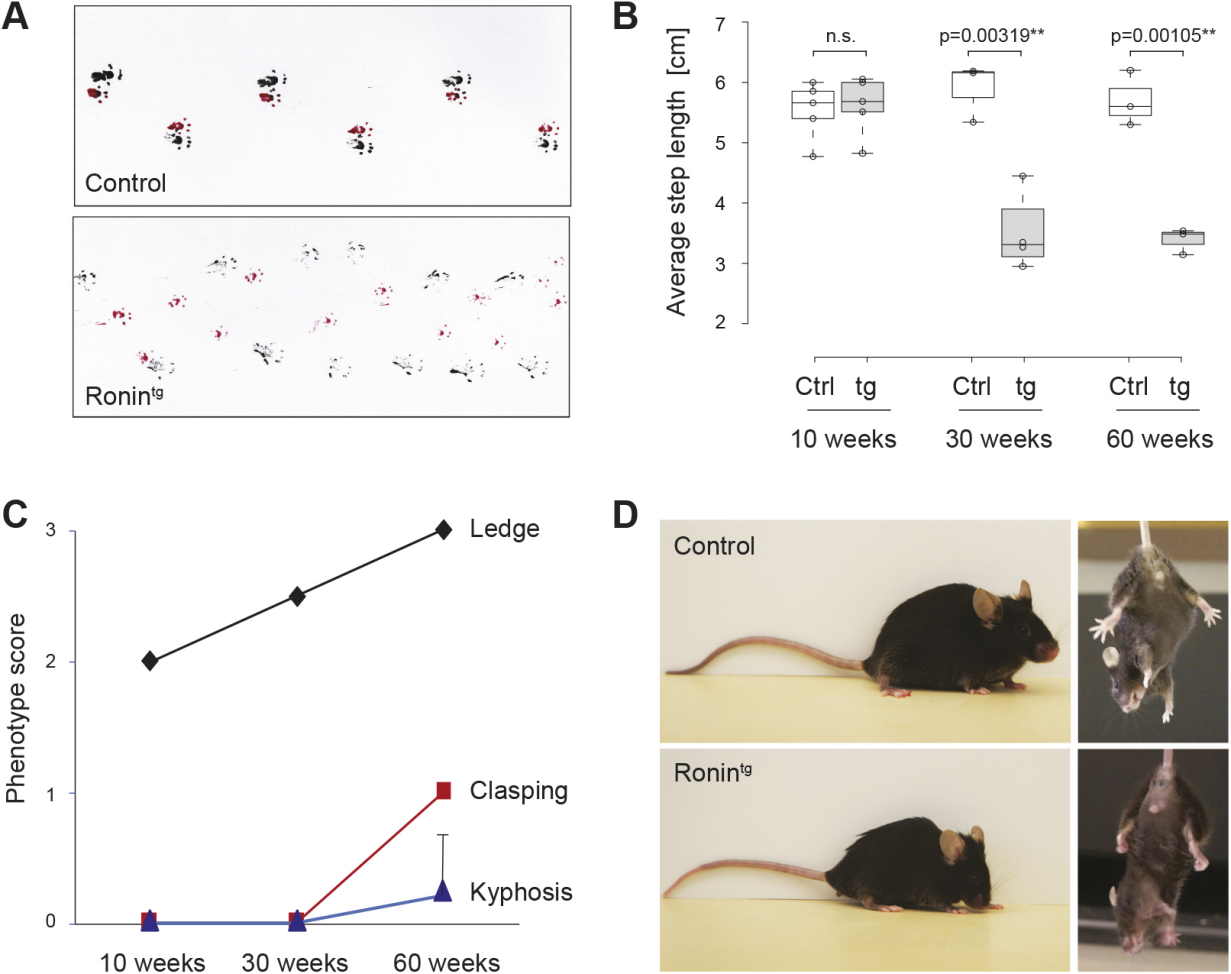
**Purkinje cell-specific transgenic expression of *Ronin* leads to ataxia.** (A) Gait pattern at 58 weeks of age. Red, front paws; black, hind paws. (B) Quantification of hindleg step length in transgenic and control animals at the indicated time points. The boxes represents the 25-75th percentiles, and the whiskers extend 1.5× the interquartile range from the 25th and 75th percentiles. The median is indicated. Each circle represents the average step length of each animal tested. *n*=5, 5, 3, 4, 3 and 3 from left to right. At least 15 steps were measured per animal. The center lines show the median. **P≤0.01; n.s., not significant (unpaired two-tailed *t*-test comparing the average values per animal between groups). Ctrl, control; tg, transgene. (C) Kyphosis, ledge performance and gait were scored between 0 and 3 according to the scoring scheme described by Guyenet et al. (2015) at the indicated time points. Data are mean±s.d. *n*=5, 7 and 7 animals per genotype per time point (10, 30 and 60 weeks, respectively). Control (wild type) littermates showed phenotype scores of 0 in all categories at all time points. (D) Typical kyphosis phenotype seen sporadically (left) and clasping behavior during a course of 20 s (right) observed in 1-year-old animals as quantified in C.

Based on these results, we focused our subsequent analyses on line C6. Transgenic animals of this line developed a marked ataxic gait (Movie 1) that led to a significantly altered footprint pattern at 30 weeks of age (Fig. 2A,B). Using an established scoring system for ataxic features (Guyenet et al., 2015), we also found a severely impaired ledge performance as early as 10 weeks that progressed with age. Hindleg clasping and sporadically occurring kyphosis were only detected after 1 year (Fig. 2C,D). As kyphosis is indicative of a non-cerebellar contribution to ataxic phenotypes, we further assessed whether transgenic animals also failed to gain weight as another measure of non-cerebellar dysfunction. However, we did not detect significant differences between transgenic and wild-type animals (Fig. S3C), suggesting that the observed phenotypes indeed mainly originated in the cerebellum.

### Purkinje cell-specific transgenic expression of Ronin leads to Purkinje cell loss

**Fig. 3.**
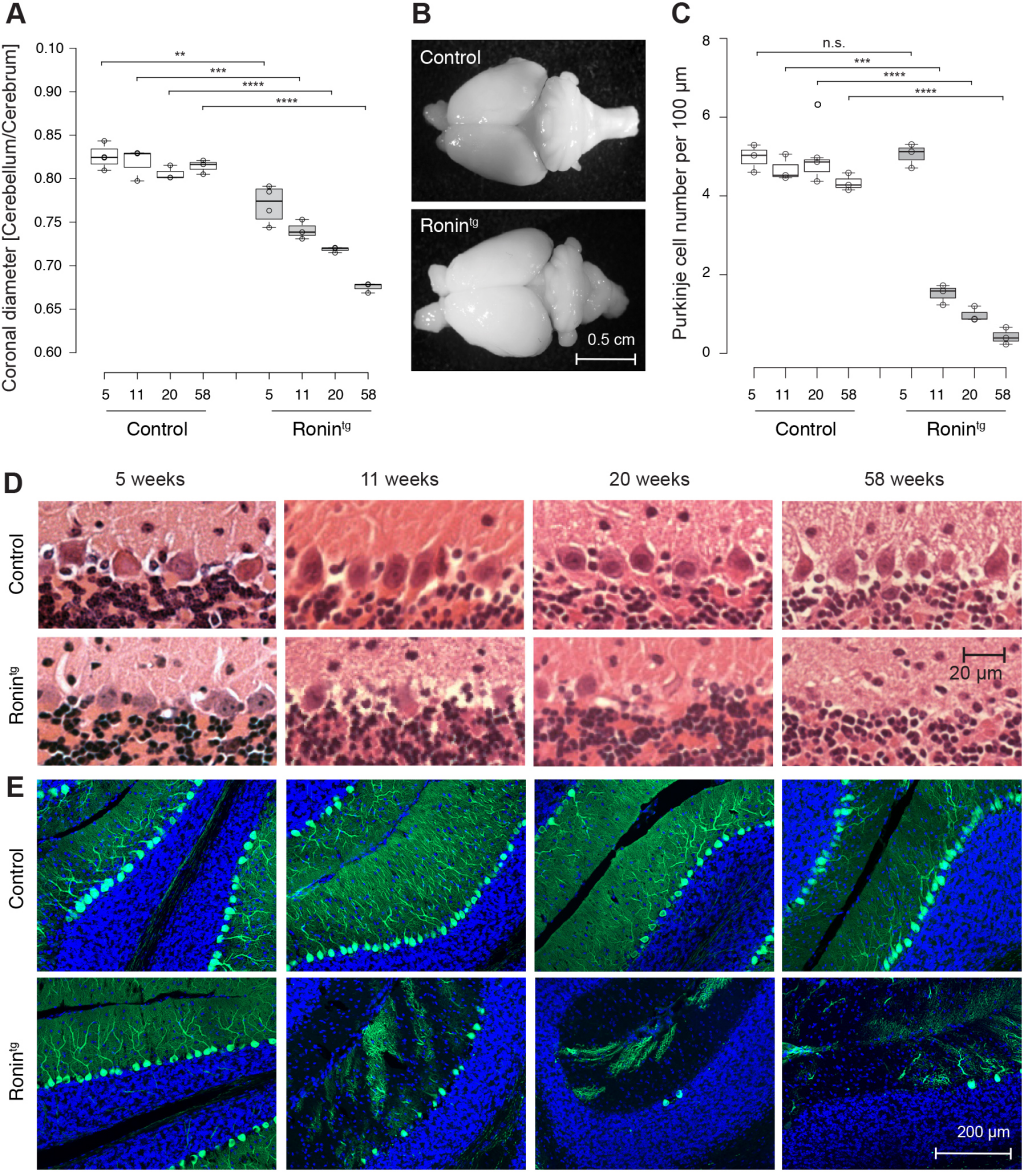
**Purkinje cell-specific transgenic expression of *Ronin* leads to loss of Purkinje cells and cerebellar degeneration.** (A) Quantification of the coronal diameter ratio between cerebellum and cerebrum at the indicated ages. Each animal is represented by a circle. *n*=4 at 5 weeks, *n*=3 at 11, 20 and 58 weeks. The center lines show the medians. *P*=5.37548E-03, 3.30734E-03, 6.34538E-05 and 1.71945E-05 from left to right. (B) Macroscopic pictures of brains isolated from 58-week-old animals as quantified in A. (C) Quantification of the number of Purkinje cells per 100 µm Purkinje cell layer in transgenic and control (wild type) animals after H&E staining of sagittal cerebellar sections. Data are presented as box plots. Each circle represents the average number of Purkinje Cells (PC) per animal; *n*=3. The boxes represent the 25-75th percentiles, and the whiskers extend 1.5× the interquartile range from the 25th and 75th percentiles. The center lines show the median. Sixty-five measures per animal across several sections of three animals per genotype at each time point were performed. *P*=0.802551867, 0.000191325, 6.33405E-05 and 2.62519E-0.5 from left to right. (D) Representative images of sagittal cerebellar sections after H&E-staining as quantified in C. (E) Immunofluorescence microscopy of cerebellar sections after staining with a specific anti-calbindin antibody was used to visualize Purkinje cells. **P≤0.01; ***P≤0.001; ****P≤0.0001; n.s., not significant (unpaired two-tailed *t*-test comparing the average values per animal between groups).

We next tested whether the movement impairments and ataxic phenotype that we observed were related to Purkinje cell loss. At 5 weeks of age, transgenic cerebella were macroscopically indistinguishable from those of their wild-type littermates (Fig. S4A), but quantification of the cerebellar to cerebral diameter nevertheless showed a slight decrease in cerebellar size (Fig. 3A). Hematoxylin and Eosin (H&E) staining of paraffin sections (Fig. 3C,D) and calbindin staining of floating cerebellar sections (Fig. 3E) at this time point revealed similar numbers, morphology and distribution of Purkinje cells in transgenic mice and wild-type littermates. The same analyses at later time points of adolescence and adulthood revealed that there was a progressive decrease in cerebellar size and Purkinje cell densities in transgenic animals compared to wild-type littermates (Fig. 3; Fig. S4). Hence, we concluded that the neuronal loss was the result of neurodegeneration and not a developmental defect. By one year of age, the cerebella of transgenic animals were substantially smaller than those of controls (Fig. 3A,B), and their Purkinje cells were largely depleted (Fig. 3C-E).

To further validate that transgenic Ronin expression itself and not the transgene integration site was responsible for the observed Purkinje cell loss, we also performed calbindin staining of cerebellar sections obtained from a 58-week-old animal of transgenic line C15. Indeed, we also found Purkinje cells to be lost (Fig. S4B). Although it is likely that the Purkinje cell loss causes the observed ataxia, we cannot exclude the more remote possibility that ectopic expression of the *Ronin* transgene in other cell types of the brain or elsewhere (Madisen et al., 2010), contributes to the described phenotype.

Because the severity of the ataxic phenotype was milder than one would expect from the major loss of Purkinje cells, we took into account that we may have overestimated the loss of Purkinje cells. Indeed, we did not scan the entire cerebella and may have overlooked regions that were less impacted. What makes this possibility less likely in our opinion is the fact that (1) the observed phenotype is progressive and reproducible, (2) the transgenic cerebella of older animals are clearly distinguishable from wild-type cerebella due to their smaller size, and (3) the severity of the ataxia is very similar in all animals.

### Purkinje cell-specific transgenic expression increases Ataxin-1 protein levels

We next addressed the question of how, mechanistically, transgenic expression of Ronin might lead to Purkinje cell degradation and ataxia. In ESCs, the function of Ronin is dependent on its co-factor Hcf1, which has been identified as a component of the ataxia network described by Lim et al. (2006). Within this network, a central role is played by the polyglutamine protein ataxin-1 (Atxn1), which is causative of one of the most common SCAs, SCA1. Atxn1 and Ronin are both proteins that bind to DNA (Dejosez et al., 2008 2010; Tsai et al., 2004) and form large protein complexes with common binding partners, such as SP1, Sin3A, Sap30 or the class 1 histone deacetylase, HDAC3 (Weissmann et al., 2018; Venkatraman et al., 2014; Dejosez et al., 2010). Furthermore, perturbation of the protein complex formed by Atxn1 and its binding partner capicua (Cic) promotes the development of SCA1 (Lim et al., 2008; Rousseaux et al., 2018), thus providing a mechanistic basis for Purkinje cell loss. Therefore, we hypothesized that Ronin/Hcf1 and Atxn1/Cic are part of the same protein complex, and that this complex might be disrupted in the cerebellum of transgenic Ronin animals.

**Fig. 4.**
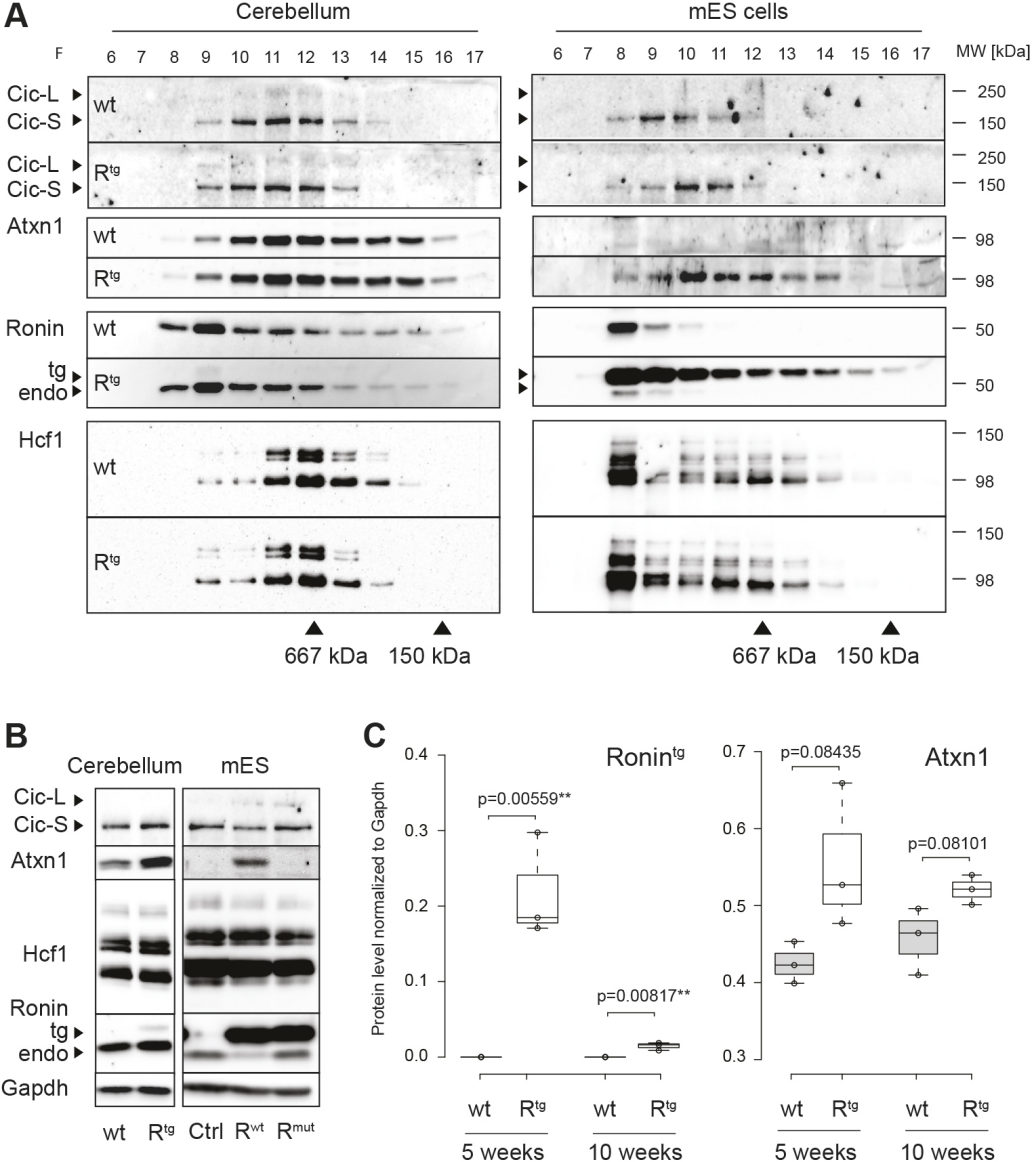
**Transgenic expression of *Ronin* induces Atxn1.** (A) Representative western blots of protein fractions collected by size exclusion chromatography of cerebellar extracts (left) or mouse ESC (mES) cell lysates (right). The size exclusion standards thyroglobulin (667 kDa) and ADH (150 kDa) are indicated. (B) Western blot analyses of equal protein amounts of cerebellar extracts from 10-week-old animals (left) or ESC lysates (right) of the indicated genotypes. (C) Quantification of transgenic Ronin (left) and Atxn1 (right) protein levels relative to GADPH, as detected by western blotting of cerebellar extracts obtained at 5 and 10 weeks of age. The boxes represent the 25-75th percentiles, and the whiskers extend 1.5× the interquartile range from the 25th and 75th percentiles. All samples are indicated as circles. Center lines show medians. **P≤0.01 (unpaired two-tailed *t*-test). wt, wild type.

To test this prediction, we performed size-exclusion chromatography and analyzed the distribution of Atxn1, Cic, Ronin and Hcf1 in cerebellar protein extracts in comparison with those of ESCs in which Ronin is known to form a large protein complex containing Hcf1 (Dejosez et al., 2008) (Fig. 4A). We found that Hcf1 peaks partially overlapped with Atxn1 and Cic peaks, but there was only marginal overlap of Hcf1 with Ronin. The latter eluted with larger complexes and had a clear distinct peak in wild-type extracts. These results suggest that Ronin and Hcf1 participate in two distinct protein complexes in the cerebellum at the time point that we analyzed (10-12 weeks). This is in contrast to the complexes found in ESCs (Fig. 4B), in which Hcf1 peaked in the same fraction as Ronin, indicating that Ronin and Hcf1 are part of the same protein complex that we observed before (Dejosez et al., 2008, 2010). In addition, and different from Ronin, Hcf1 had a second elution peak in fractions similar to those in which it eluted in cerebellar extracts. However, overall, our data suggest that Ronin-Hcf1 and Atxn1-Cic do not cooperate in the same complex. In line with these results, we were unable to detect any interaction of Ronin with Atxn1 or Cic in immunoprecipitation studies using an anti-Flag antibody in ESC extracts (Fig. S5A). Meanwhile, the previously described binding of Hcf1 to Ronin was readily visible (Dejosez et al., 2008).

Although we did not find any differences in the distribution of any of the analyzed proteins between wild-type and transgenic samples per se, we noticed an increase in Atxn1 protein levels in EF1α-Ronin-Flag ESCs (Fig. 4A,B) compared with controls. Indeed, Atxn1 was completely undetectable in control ESCs but emerged in Ronin-overexpressing cells. This induction was dependent on Hcf1 as expression of a Ronin mutant incapable of binding to Hcf1 (Dejosez et al., 2010) did not show any Atxn1 protein (Fig. 4B, right) just like control cells. Therefore, Ronin is a potent inducer of Atxn1 *in vitro* and, importantly, Ronin also induced Atxn1 protein levels in our transgenic *in vivo* model (Fig. 4B). Quantification of the protein levels (Fig. 4D; Fig. S5B) confirmed a subtle induction of Atxn1 in cerebella of 5-week-old transgenic animals compared to wild-type littermates. The induction was still detectable at 10 weeks of age but to a lesser extent, which could be attributed to the severe loss of Purkinje cells that is also reflected in the substantial reduction of transgenic Ronin protein at this time point (Fig. 4C; Fig. S5C). Notably, the induction was not significant, which might be attributed to the fact that Atxn1 is also expressed in other cerebellar cell types that are not affected in our Purkinje cell model. We also found that the cerebellar levels of endogenous Ronin decreased slightly with age in wild-type animals, whereas this trend was not seen in transgenic animals (Fig. S5B,C). A possible explanation for this apparent lack of responsiveness of the endogenous gene in transgenic animals might be a compensatory feedback loop induced within the remaining Purkinje cells or other surrounding cells. Alternatively, the increase in Ronin signal may simply reflect a change in cell composition of the transgenic cerebellum.

The induction of Atxn1 was an interesting finding because studies have shown that an increase of wild-type Atxn1 protein can induce ataxia in mice even in the absence of a polyQ expansion through a gain of function of the Atxn1-Cic complex (Gennarino et al., 2015; Fernandez-Funez et al., 2000). However, because the increase of Atxn1 observed in our system is subtle, it cannot explain the severe loss of Purkinje cells seen at an early age in our mouse model.

### Ronin overexpression alters the cerebellar transcriptome

**Fig. 5.**
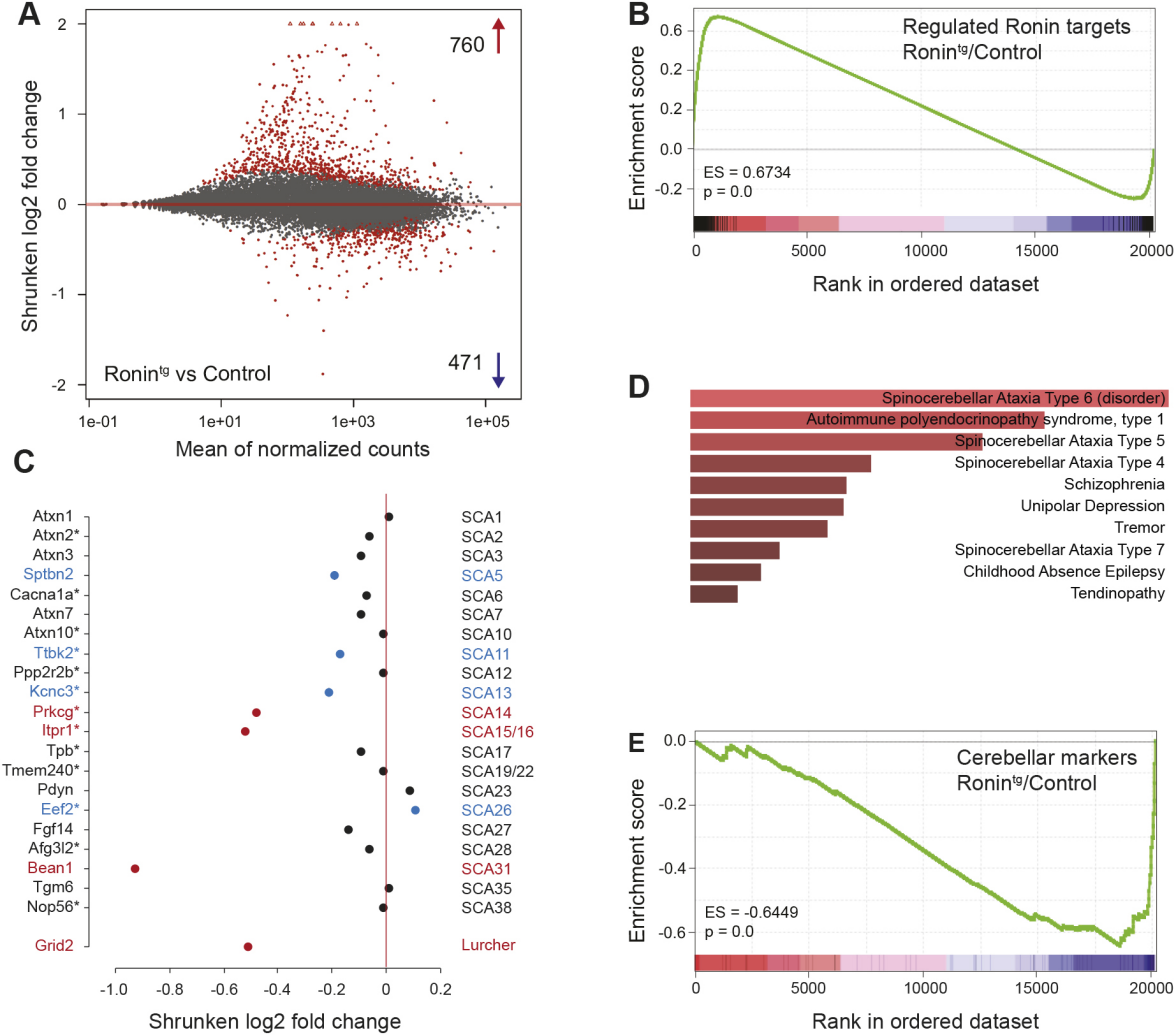
**RNA-seq analyses of L7-Ronin^tg^ animals at 5 weeks of age.** (A) MA plot of RNA-seq data showing the log-fold change (M-value) against the log-average (A-value). Points are colored in red if the adjusted *P*-value was less than 0.1. Points that fall outside the window are plotted as open triangles. (B) GSEA of Ronin targets that are differentially expressed in Ronin^tg^ cerebella compared with wild-type control cerebella. (C) Expression of genes (indicated on the left) that have been identified to cause SCAs (indicated on the right), as well as Grid2, the loss of which causes the Lurcher phenotype in mice. Genes that are Ronin targets in ESCs are marked with an asterisk; genes that are differentially expressed with *P*≤0.5 are marked in blue; those with an adjusted *P*-(padj)≤0.1 are highlighted in red. (D) Top categories enriched for genes that were downregulated ≤1.25-fold in Ronin^tg^ cerebella versus wild-type controls, sorted by their *P*-values. (E) GSEA of cerebellar markers in Ronin^tg^ cerebella compared with wild-type control cerebella.

To gain further insight into the mechanism by which Ronin overexpression leads to Purkinje cell loss, we performed RNA-seq analyses of Ronin^tg^ and wild-type control cerebella isolated from 5-week-old animals. This allowed us to survey the transcriptional changes at a time point at which we did not detect a difference in the Purkinje cell number (Fig. 5A; Table S1A). Among the genes that were altered by more than 1.25-fold, we observed positive enrichment of Ronin targets that are known to be bound by Ronin in ESCs (Fig. 5B).

Gene ontology (GO) analysis of this upregulated Ronin target gene set revealed the enrichment of genes related to wounding, response to stress and programmed cell death (e.g. caspase-3), among other categories (Table S1B). When we specifically looked at all genes that have been identified to cause SCAs (Fig. 5C), we found that many of them contained Ronin DNA-binding motifs and were bound by Ronin (Dejosez et al., 2010). Several of them were downregulated, some significantly. Interestingly, we did not detect an alteration of Atxn1 RNA level in the cerebellum despite the slight elevation detected in Ronin-overexpressing ESCs (Fig. S5D). This may have been because of our use of the entire cerebellum; high expression of Atxn1 in other regions of the cerebellum may mask an increase in Purkinje cells. It is noteworthy that expression of Grid2, which causes the Lurcher phenotype in mice, characterized by rapid extensive Purkinje cell loss (Caddy and Biscoe, 1976), was also among the downregulated genes.

When we used the EnrichR tool to search the DisGeNet database (a collection of human disease-associated genes) with the set of genes that were downregulated more than 1.25-fold (Table S1C), we found that SCA6-, 5-, 7- and 2-related genes were enriched among the top categories with the highest *P*-values (Fig. 5D). It remains unclear whether the changes were direct transcriptional effects or secondary in nature, and we cannot tell whether they originated in Purkinje cells or in surrounding cells. However, it is likely that Ronin, a transcriptional regulator of housekeeping genes, induces transcriptional changes that are not tolerated by the Purkinje cells. Our analysis suggests that even though the Purkinje cell number is not altered at this early time point (5 weeks), the cells appear to be damaged already. This observation was verified by the negative enrichment of cerebellar markers (Lein et al., 2007) in transgenic versus wild-type control animals in gene set enrichment analyses (GSEA) (Fig. 5E), which conforms with the slight decrease in cerebellar size we measured at this time point (Fig. 3A).

## DISCUSSION

We have shown that an increase in Ronin activity in cerebellar Purkinje cells leads to Purkinje cell loss and the development of severe ataxia in mice as early as 10-12 weeks after birth. Our data suggest that Ronin might be involved in the development of ataxia, including SCA4 and other neurodegenerative diseases, not through classical mutations in the gene body itself but rather through genetic defects that affect its activity (e.g. mutations in regulatory elements or CNVs). At the mechanistic level, our data show deregulation of critical Ronin target genes at an age at which the Purkinje cell number is not altered, and further suggest that direct transcriptional perturbation is most likely to cause the degradation of Purkinje cells. We also found that transgenic Ronin expression upregulates Atxn1, the causative gene of SCA1. This was an important finding as it introduces Ronin as a positive regulator of Atxn1, and recent studies have shown that an increase of wild-type Atxn1 protein leads to a gain of function of the Atxn1-Cic complex, which is sufficient to induce ataxia in mice, even in the absence of a polyQ-expanded Atxn1 (Gennarino et al., 2015; Fernandez-Funez et al., 2000). Because Ronin and its co-factor Hcf1 are associated with transcriptional regulation, the most parsimonious explanation for the increased Atxn1 levels in the presence of transgenic Ronin expression would be that Ronin exhibits transcriptional control over *Atxn1*. Although *Atxn1* mRNA levels were indeed higher in *Ronin* overexpressing ESCs compared to control cells (Fig. S5C; Dejosez et al., 2010), we did not detect such an increase in RNA level in the cerebellum. Hence, it remains to be determined whether Ronin affects *Atxn1* transcription through direct gene regulation or indirectly by transcriptional regulation of genes involved in stabilizing or destabilizing *Atxn1* mRNA, such as Pumilio (Pum1). Pumilio is known to destabilize *Atxn1* mRNA, and its haploinsufficiency increases Atxn1 protein levels causing ataxia in mice (Gennarino et al., 2015). The effect could also be explained by the direct or indirect modulation at the post-transcriptional level, such as Atxn1 phosphorylation. However, we only observed a subtle induction of Atxn1, and overexpression of wild-type Atxn1 does not cause ataxia and Purkinje cell loss to the extent we see in our Ronin transgenic mouse line (Fernandez-Funez et al., 2000). Hence, we concluded that the increased Atxn1 levels seen in our system could not explain the observed phenotype, which is most likely a combinatorial result.

Alternatively, Ronin may deregulate critical target genes through different molecular means altogether. For instance, its binding has been associated with 5-hydroxy-methylcytosines, and this finding links Ronin activity to the DNA methylation status at promoters (Spruijt et al., 2013). The DNA recognition sequence of Ronin is frequently found in chromatin loop anchors (Bailey et al., 2015), making it conceivable that some of its actions may be exerted through changes in the three-dimensional genomic organization of promoters. The gene regulation of Ronin and its DNA recognition sequence have been critically linked to heart development (Fujita et al., 2017), retina development (Poche et al., 2016) and disease (Radziwon et al., 2017), as well as cobalamin metabolism (Quintana et al., 2017). Additionally, polyglutamine proteins, such as Ronin, have recently been associated with the assembly of functional units (e.g. Pol II) through a biophysical process called phase separation (Toretsky and Wright, 2014). Given the DNA-binding ability of Ronin, its overexpression could alter gene expression by modulating Pol II recruitment. It is also noteworthy that Ronin was discovered as a caspase-3 target that has a functional caspase-3 cleavage site following the polyglutamine repeat (Fujita et al., 2017). Caspase cleavage products have been shown to contribute to the development of SCAs (Berke et al., 2004; Liman et al., 2014; Guyenet et al., 2015) and other neurological disorders (Graham et al., 2006). Thus, in light of the induction of caspase-3 activation we observed at the transcriptional level (Table S1), it is possible that caspase-3-mediated cleavage might lead to the accumulation of Ronin fragments, which could contribute to the ataxic phenotype. Finally, Ronin was recently identified as a direct negative regulator of Parkin (Park2), which causes a familial form of Parkinson's disease (autosomal recessive juvenile) when mutated (Potting et al., 2018). Park2 is also known to interact with Ataxin 2 (Atxn2), the polyglutamine expansion of which causes SCA2. As Park2 regulates the intracellular levels of both wild-type and mutant Atxn2, and inhibits Atxn2-induced cytotoxicity (Huynh et al., 2007), elevated Ronin protein levels could lead to accumulation of Atxn2 through repression of Park2. Further detailed studies are needed to test whether these effects are at play, and if they are due to direct or indirect transcriptional control by Ronin.

It is remarkable that gene dosage changes of *Ronin* in its wild-type form can have such detrimental effects on neurons and induce such severe pathological outcomes. Given the robustness of the observed ataxia phenotype, it will be interesting to see whether Ronin does indeed prove to be involved in 16q22.1-linked SCA4. In this case, we predict that genomic rearrangements (e.g. CNVs) or non-coding mutations that affect Ronin levels might explain SCA4 disease and possibly other 16q22.1-linked ataxias. Such a scenario would add to the list of genomic rearrangement-related disorders, such as Charcot-Marie-Tooth disease (Parker et al., 2015), which is characterized by CNVs, SCA2, where the increased gene dosage in patients homozygous for the disease-related genetic defect influences the age of disease onset (Spadafora et al., 2007), autosomal dominant early-onset Alzheimer's disease caused by a duplication of the amyloid precursor protein locus (Rovelet-Lecrux et al., 2006; Rumble et al., 1989), and familial Parkinson's disease associated with duplications or triplications of α-synuclein (SNCA) (Chartier-Harlin et al., 2004; Ibáñez et al., 2004; Singleton et al., 2003). Alternatively, simple mutations in the regulatory elements of *Ronin* (e.g. in one of its enhancers or the promoter) could cause chronic or context-specific misexpression to contribute to the disease. In light of the genetically stable CAG repeat of *Ronin* and the absence of polyQ conglomerates in SCA4 patients (Hellenbroich et al., 2006), our results suggest that such patients should be screened for duplications of the *Ronin* gene and possible mutations within the Ronin-binding motif of Ronin-bound promoters within the genomic region mapped to SCA4.

Future studies are needed to clarify whether expansion of the polyQ region in Ronin might lead to more severe outcomes. CAG repeat length differences have been described in both normal individuals and patients with neurological disorders, ranging from 10-41 (Yin et al., 2014; Pandey, et al., 2004; Butland et al., 2007). A repeat length of 29 is the most frequent (∼70%) followed by 28 repeats (∼20%), with no significant difference between normal and diseased individuals. Interestingly, a 38Q repeat was detected in two neurodegenerative disease patients, and expansion of the glutamine repeat *in vitro* leads to increased formation of nuclear aggregates in PC12 cells (Yin et al., 2014). Given the ataxic phenotype in our mouse model, we predict that further analyses of human Ronin, its binding partners and its binding sites in target gene promoters might reveal novel mutations in the spectrum of neurodegenerative disease.

## MATERIALS AND METHODS

### Generation of animals with Purkinje cell-specific transgenic expression of Ronin

To direct Ronin expression specifically to the Purkinje cells, a Not1/Sal1 fragment of pEF1α-hRonin-Flag-Ires-Neo (Dejosez et al., 2008), containing the human *Ronin* coding sequence, fused to a C-terminal Flag-tag, was cloned by blunt-end ligation into the unique BamHI site of the pL7-AUG-EcoRI vector, which is located in the fourth exon of the *Pcp2*/*L7* gene (Smeyne et al., 1995). The vector includes ∼1 kb of the promoter, the four exons and three introns, as well as ∼200 bp upstream sequence of the *Pcp2*/*L7* gene. After sequence verification and HindIII digest, the construct was injected into single-cell blastocysts, which were transplanted into C57BL/6 foster animals by the Genetically Engineered Mouse Core at Baylor College of Medicine. All experimental procedures and protocols were approved by the Institutional Animal Care and Use Committee of Baylor College of Medicine (AN-4011) or the Icahn School of Medicine at Mount Sinai (IACUC-2013-1433). Founder animals were backcrossed to C57BL/6 animals to establish independent lines. L7-Ronin-Flag transgenic animals were identified by PCR of tail biopsies. DNA was isolated from 1-2 mm of tail tissue using a DNeasy Blood and Tissue Kit (Qiagen) following the protocol for isolation of DNA from animal tissues, and was eluted in 100 µl H_2_O. Subsequently, 2 µl DNA was used as template in two transgene-specific PCR assays. The PCR reaction was performed using GoTaq Green Mastermix (Promega) in a total volume of 50 µl in the presence of 0.2 µM of each oligo. The L7-specific 5′ oligo MAD041 (5′-TGTTTGGAGGCACTTCTGACTTGC-3′) and the transgene-specific oligo MAD224 (5′-CTGACTGCTGTCTACAGTGGCCTG-3′) amplified a 578 base pair fragment, whereas the 5′ transgene-specific oligo MAD225 (5′-GCGGCCGCAAGACCTACACGGTACG-3′) and the 3′ L7-specific oligo MAD042 (5′-CACTCAACTCTTTGTTGCTAGTGC-3′) resulted in a 969 base pair fragment. The following cycle conditions were used: denaturation for 3 min at 94°C; 40 cycles of 30 s at 94°C; 30 s at 55°C and 30 s at 72°C; followed by a final extension at 72°C for 7 min. Animals of both genders were included in this study. Controls were age and gender matched if not otherwise stated.

### Southern blot analysis

To determine the copy number of the integrated transgene, Southern Blot analysis was performed as described previously with a *Ronin*-specific probe complementary to part of the C terminus (Dejosez et al., 2008).

### RNA isolation and RT-PCR from cerebellar tissue samples

Cerebella were collected from 5-week-old animals and subjected to Dounce homogenization. RNA from individual cerebella was isolated after homogenization with a Lipid Tissue RNA Kit (Qiagen) following the manufacturer's instructions. RNA (1 µg) was used to create cDNA with an ImpromII Reverse Transcriptase Kit (Promega) following the manufacturer's standard protocol, including oligodT oligos and 4.8 M MgCl_2_ in each 20 µl reaction (Promega). Each reaction (1 µl) was used as template in subsequent PCR amplification reactions in a final volume of 50 µl with GoTaq Green Mastermix (Promega) in the presence of 0.2 M of each oligo under the following conditions: denaturation at 94°C for 3 min; 30 cycles of 94°C for 30 s; 55°C for 30 s; and 72°C for 30 s; followed by a final extension of 72°C for 7 min. To detect the *hRonin*-Flag transgene mRNA the oligos MAD041 and MAD224 (see above) were used, whereas Actb mRNA was detected with the oligos MAD233 (5′-GGCCCAGAGCAAGAGAGGTATCC-3′) and MAD234 (5′-ACGCACGATTTCCCTCTCAGC-3′), resulting in PCR fragments of 578 and 466 base pairs, respectively.

### Rotarod analysis

Motor function was analyzed on a rotarod (Ugo Basile) accelerating from 4-40 rpm for 300 s, and latency to fall was recorded in four consecutive trials on three consecutive days (Watase et al., 2002). All animals were from the second generation. Animals in each group were born in a range of 2 weeks. Females and males were included in each group. *P*-values were calculated using mixed effect hierarchical models. The animals of each mouse line at 10-12 or 20-22 weeks of age were treated as random effects and nested within the repeat experiment (T1-T4) and day (day 1-day 3). Genotypes, repeat experiments and days were considered fixed effects in the models. Orthogonal contrasts within the models were used to compare the genotypes at different points. These contrasts standard errors are based on the model covariance matrix. We did not correct for the multiplicity of the contrast as the *P*-value for the difference between days should be considered as descriptive, and the *P*-values for the comparisons across days should be considered for all separate experiments.

### Footprint analysis

Footprint analysis was performed as described previously (Chen et al., 2012). Briefly, forepaws and hindpaws were painted with non-toxic red or black paint. The animals were allowed to walk through a tunnel lined with a sheet of paper. The average hindleg step lengths of at least three wild-type and three transgenic animals per time point were determined by measuring the length of at least 15 steps per animal.

### Calbindin and Ronin staining of floating cerebella sections

Immunofluorescence staining of floating sections after perfusion was performed as described previously (Baader and Schilling, 1996). Briefly, the animals were anesthetized and perfused with 4% paraformaldehyde (PFA) for 3 min. The brain was dissected, further fixed in 4% PFA for 16 h and then treated in 20 ml of 5%, 10%, 15%, 20% and 25% sucrose for at least 1 h or until the tissue sank to the bottom of the reaction tube. The brain tissue was cut in half and frozen in optimal cutting temperature (OCT) medium. Sections (50 µm) were cut and OCT was removed by two washes in PBS. Sections were blocked for 1 h in blocking solution (2% normal goat serum and 0.3% Triton X-100 in Dulbecco's PBS), followed by incubation at 4°C in a 1:500 dilution of the anti-calbindin-D-28K antibody (Sigma-Aldrich, CB-955) in blocking solution for 48 h. Sections were washed four times for 20 min at room temperature and incubated with a 1:500 dilution of the goat anti-mouse IgG Alexa-Fluor 488 antibody (Molecular Probes, A11029) in blocking solution for 48 h at 4°C. The sections were washed four times at room temperature for 20 min, rinsed in PBS, transferred to microscope slides in 2% bovine serum albumin solution, dried and mounted in ProLong Gold with DAPI. Animals of both genders were analyzed and compared to age- and gender-matched controls. Ronin staining was performed using a 1:100 dilution of directly PE-conjugated monoclonal anti-Ronin antibody (Becton Dickinson, 562549) following the same protocol, but omitting the secondary antibody staining step. Imaging was performed using structured illumination microscopy with an AxioImager Z2M with ApoTome (Zeiss), and the images were processed using ZEN software (Zeiss).

### Hematoxylin and Eosin staining

Cerebella were fixed after perfusion, embedded in paraffin and 10 μm sections were stained with H&E by the Histology Service of the Department of Pathology at the Baylor College of Medicine.

### Cell culture

EF1α-Ires-Neo, EF1α-Ronin-Flag-Neo and EF1α-Ronin^DHSA^-Flag-Neo (Ronin^mut^) ESCs were cultured as described previously (Dejosez et al., 2010). We established stocks of all cell lines with genotypes that were verified by PCR and sequencing of the PCR products.

### Column fractionation

Complex purification and western blot analyses were performed as described previously (Lam et al., 2006). Briefly, chromatography was carried out at 4°C using the GE AKTA Purifier UPC 10 FLPC system. Cellular or cerebellar extracts were prepared fresh by Dounce homogenization of ∼25 million ESCs or two cerebella from age-matched mice (10-12 weeks) in TST buffer [50 mM Tris (pH 8), 75 mM NaCl, 0.5% Triton X-100, 1 mM PMSF and protease inhibitors]. An aliquot of 700 µl was loaded for fast protein liquid chromatography. Gel filtration was performed using a Superose 6 10/300 GL column (GE Healthcare) equilibrated in buffer [50 mM Tris (pH 8), 50 mM NaCl and 0.1% Triton X-100] at 0.3 ml/min. Fractions were collected every 0.5 ml or 1 ml volume, column void volume was 7.7 ml, and elution volumes of gel-filtration standards were 12.4 ml for thyroglobulin (669 kDa), 15.8 ml for alcohol dehydrogenase (150 kDa), and 19.2 ml for cytochrome C (12.4 kDa). Anion exchange was performed using a MonoQ 5/5 HR column (GE Healthcare) equilibrated in buffer [50 mM Tris (pH 8), 50 mM NaCl and 0.1% Triton X-100]; after washing the column with 13 column volumes of the same buffer, bound proteins were eluted with a 20 ml linear NaCl salt gradient from 50 mM to 600 mM NaCl in 50 mM Tris (pH 8) and 0.1% Triton X-100 at 1 ml/min; 1 ml fractions were collected. All fractions were supplemented with protease inhibitors and immediately prepared for SDS-PAGE.

### Protein analyses by western blot

SDS-PAGE and western blotting were performed using standard procedures (Chen et al., 2003; Watase et al., 2002). The following antibodies and dilutions were used: guinea pig polyclonal anti-Cic serum (Lam et al., 2006, 1:2000); mouse monoclonal anti-FLAG M2 (Sigma-Aldrich, F7425, 1:1000); rabbit polyclonal anti-Atxn1 (Lam et al., 2006, 11750VII, 1:5000); mouse monoclonal anti-GAPDH (Advanced Immunochemical, 2-RGM2, 1:20,000); mouse monoclonal anti-Ronin (Becton Dickinson, 562548, 1:1000); rabbit polyclonal Hcf1 (Bethyl Laboratories, A301-400A, 1:1000); goat anti-mouse-horseradish peroxidase (Jackson Lab, 115-035-003, 1:50,000); goat anti-guinea pig-HRP (Jackson Lab, 106-035-003, 1:8000); and goat anti-rabbit-HRP (Bio-Rad, 1706515, 1:100,000). Signal quantification was performed using Image J (version 1.51, National Institutes of Health). *P*-values were calculated using two-tailed *t*-tests.

### RNA-seq

RNA isolation from the cerebella of 5-week-old mice and RNA-seq analyses were performed using Active Motif. Briefly, 42-base pair sequence reads were generated using an Illumina NextSeq 500 and mapped to the mouse genome using the STAR algorithm with default settings. After obtaining the gene table containing the fragment (or read) counts of genes, differential analysis using DEeq2 was performed to identify genes that showed significant differences. After a differential test was applied to all genes with non-zero counts, the *P*-value of each gene was calculated, and multiple-testing adjustment was performed. Differential genes were detected at a false discovery rate of 0.1 (or 10%; i.e. adjusted *P*-value).

### Gene set enrichment, gene ontology and EnrichR DisGeNet analyses

GSEA was performed using the GSEAPreranked module of the GenePattern online tool (https://cloud.genepattern.org) with the recommended parameters (1000 permutations, weighted scoring scheme, Max_probe collapsing mode for probe sets with more than one match, meandiv normalization mode). The GO enrichment analysis was performed using ShinyGO v.061 (http://bioinformatics.sdstate.edu/go/). The EnrichR online tool (https://amp.pharm.mssm.edu/Enrichr/) was used to search the DisGeNet database, which is a collection of human disease-associated genes.

### Data illustration and statistical analysis

Box plots were generated using BoxPlotR (http://shiny.chemgrid.org/boxplotr/). Center lines show the medians; box limits indicate the 25th and 75th percentiles; whiskers extend 1.5 times the interquartile range from the 25th and 75th percentiles; and outliers are represented by circles. *P*-values were calculated using a *t*-test when not specified. When several values per animal were measured, the *P*-value was based on the average value per group that was calculated from the average value per animal.

## Supplementary Material

Supplementary information
