# Supplementary material for: Ronin overexpression induces cerebellar degeneration in a mouse model of ataxia
Source: Dis Model Mech. 2021 Jun 24;14(6):dmm044834. doi: 10.1242/dmm.044834 (PMC8246265; doi:10.1242/dmm.044834)
Supplement: Supplementary information [file dmm-14-044834-s1.pdf]

A

```
human   MPGFTCCVPGCYNNSHRDKALHFYTFPKDAELRRLWLKNVSRAGVSGCGSTGQPTTGHRLCSVHF
mouse   MPGFTCCVPGCYNNSHRDKALHFYTFPKDAELRRLWLKNVSRAGVSGCGSTGQPTTGHRLCSVHF
        *****************************************************************

human   QGGTKTYTVRVPTIFPLRGVNERKVARRPAGAAAARRRQQQQQQQQQQQQQQQQQQQQQQQQQQQ
mouse   QGGTKTYTVRVPTIFPLRGVNERKVARRPAGAAAARRRQQQQQQQQQQQQQQQ---------LQQ
        *****************************************************          **

human   QQSSPSASTAQTAQLQPNLVSASAAVLLTLQATVDSSQAPGSVQPAPITPTGEDVKPIDLTVQVE
mouse   QQPSPSSSTAQTTQLQPNLVSASAAVLLTLQAAVDSNQAPGSVVPVSTTPSGCCVKPIDLTVQVE
        **.***:*****:*******************:***.****** *.. **:*: ***********

human   FAAAEGAAAAAAAASELQAATAGLEAAECPMGPQLVVVGEEGFPDTGSDHSYLSSGTTEEELLRK
mouse   FAAAEGAAAAAAAASELEAATAGLEAAECTLGPQLVVVGEEGFPDTGSDHSYLSSGTTEEELLRK
        *****************:***********.:*********************************

human   LNEQRDILALMEVKMKEMKGSIRHLRLTEAKLREELREKDRLLAMAVIRKKHGM 314
mouse   LNEQRDILALMEVKMKEMKGSIRHLRLTEAKLREELREKDRLLAMAVIRKKHGM 305
        ******************************************************
```

B

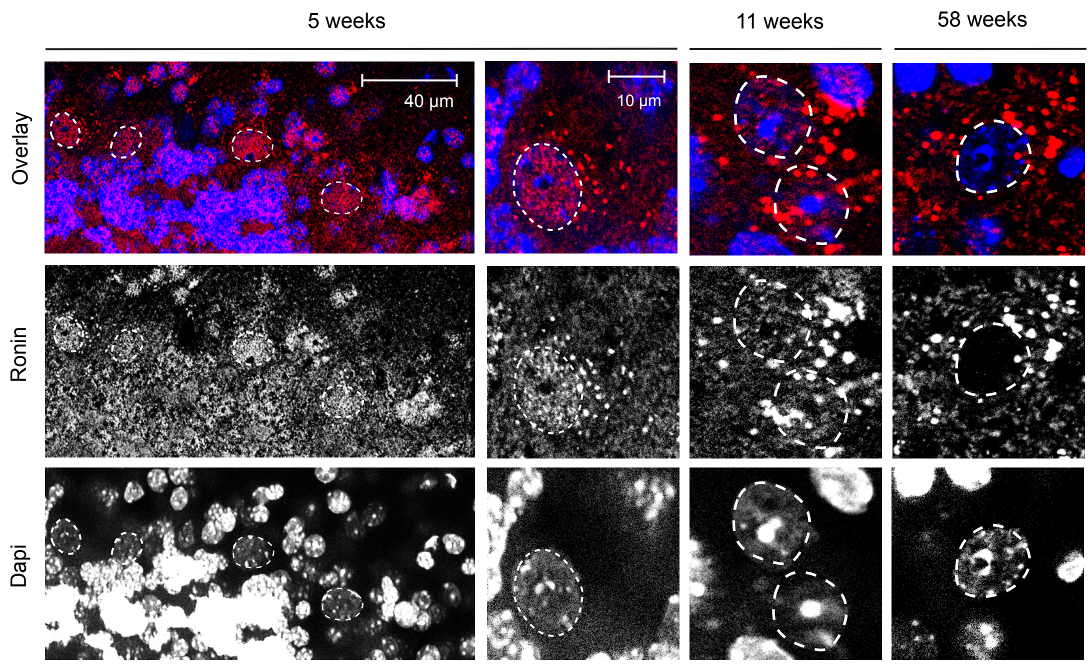

C

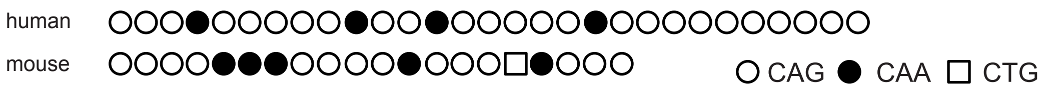

Fig. S1. Alignment of mouse and human Ronin protein sequence and Ronin expression in Purkinje cells. (**A**) The mouse and human Ronin protein sequences are nearly identical at the C- and N-termini but differ in the polyglutamine region that separates these domains. (**B**) Immunofluorescence staining of floating cerebellar sections from wildtype animals at 5, 11 or 58 weeks of age with a directly PE-conjugated anti-Ronin antibody. (**C**) The polyglutamine-coding regions differ between mouse and human Ronin. The human sequence contains 29 glutamine-coding codons while the mouse sequence has a shorter repeat of 21 codons. Both are considered relatively stable repeats as the CAG codons (open circle) are interrupted by CAA (dark circle) or CTG (square) codons.

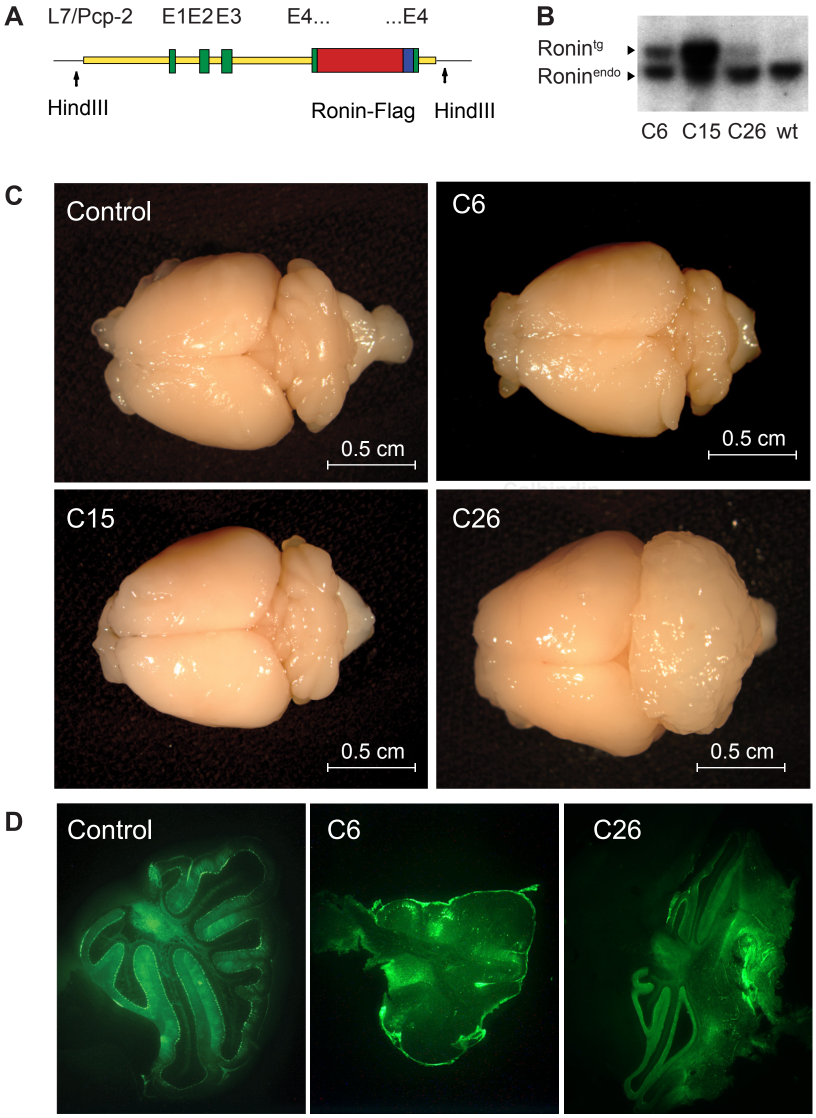

Fig. S2. Schematic of the Pcp2/L7-Ronin construct and phenotype of transgenic founder animals. (**A**) Schematic illustration of the Pcp2/L7-*Ronin* construct. C-terminally Flag-tagged human *Ronin* was integrated into the fourth exon of Pcp2/L7 within the pL7-AUG-EcoRI vector. (**B**) Southern blot analysis of transgenic animals in comparison to a wildtype (wt) control animal using a probe that detects the L7-Ronin transgene ($Ronin^{tg}$) and endogenous Ronin ($Ronin^{endo}$). (**C**) Morphology of brains isolated from transgenic founder and wildtype control animals at 36 weeks of age. (**D**) Calbindin staining of sagittal cerebellar sections from founders C6 and C26 in comparison with an age- matched wildtype control animal at 36 weeks of age. endo, endogenous; tg, transgene.

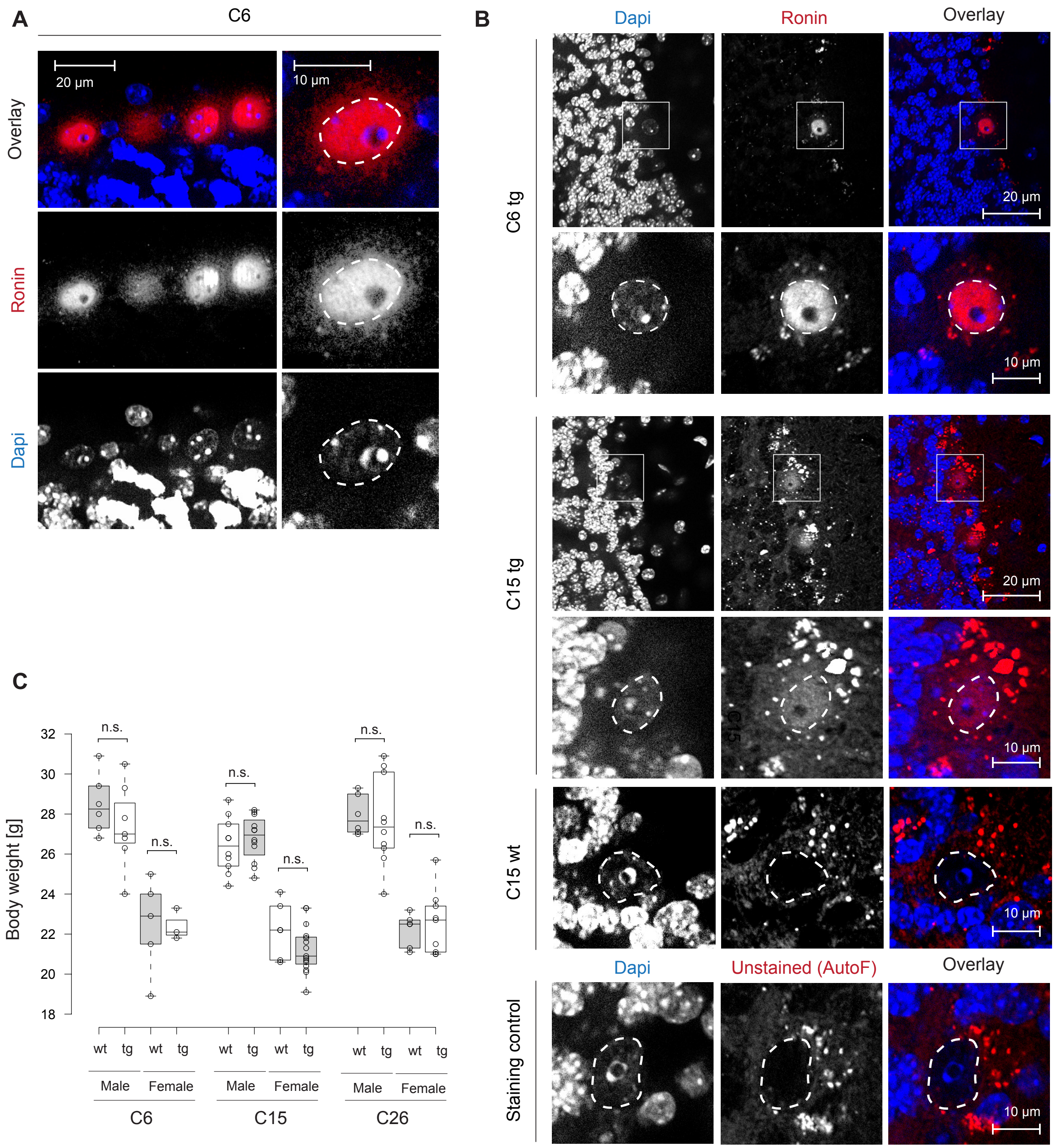

Fig. S3. Purkinje cell-specific transgenic expression of Ronin does not affect weight. (A) Immunofluorescence staining of floating sagittal sections of cerebella isolated from a 5-week-old transgenic C6 animal and (**B**) Immunofluorescence staining of cerebellar sections obtained from lines C6 (58 weeks) or C15 (59 weeks) with a directly PE-labeled Ronin-specific antibody. A wildtype control littermate of line C6 of the same age is shown in Figure S1B. (**C**) Weight of male and female animals from transgenic lines C6, C15 and C26 at 20-22 weeks of age. Each animal is represented by a circle; center lines represent the medians of each group. p-values were calculated by t-test. AutoF, autofluorescence; n.s., not significant; tg, transgene, wt, wildtype;

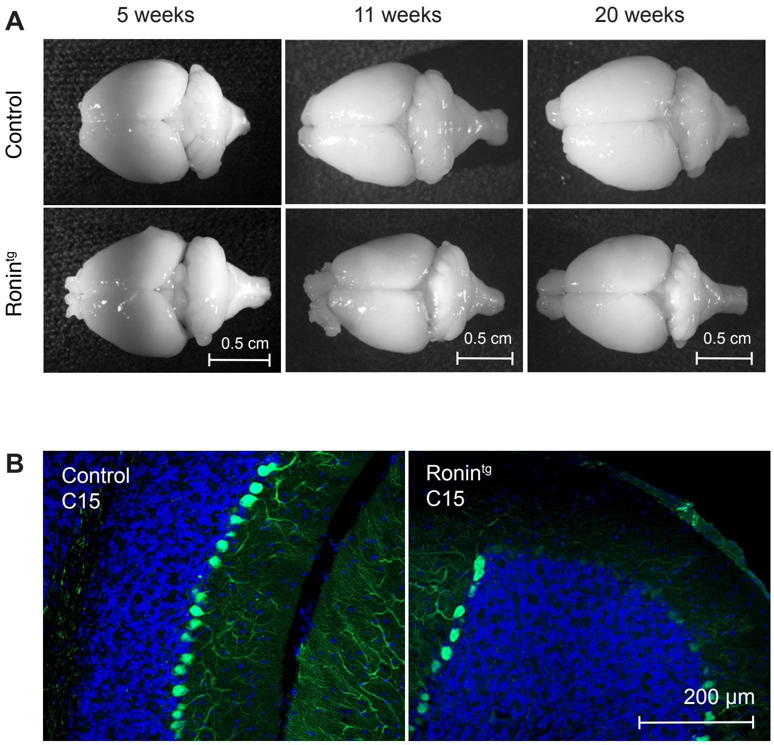

Fig. S4. Purkinje cell-specific transgenic expression of Ronin leads to loss of Purkinje cells and cerebellar degeneration. (**A**) Macroscopic pictures of brains isolated from animals of line C6. (**B**) Immunofluorescence staining of floating sagittal cerebellar sections of 58-week-old animals from line C15 (exhibiting the lowest Ronin^{tg} transcript levels) with an anti-Calbindin antibody to visualize Purkinje cells. tg, transgene.

Disease Models & Mechanisms • Supplementary information

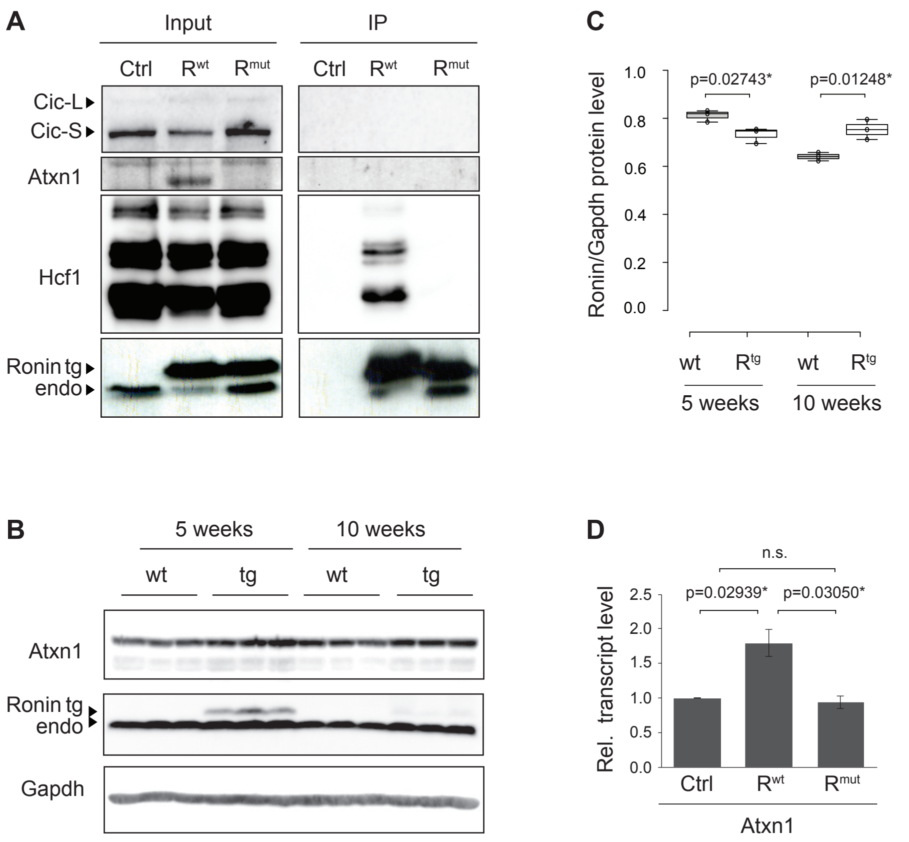

Fig. S5. Transgenic expression of Ronin increases Atxn1 protein levels. (**A**) Immunoprecipitation with a Flag antibody using protein extracts prepared from ES cells overexpressing Luciferase (Ctrl, control), wildtype Ronin (R^{wt}) or a Ronin mutant (R^{mut}) incapable of binding to Hcf1, followed by detection of Cic or Atxn1. (**B**) Western Blot analyses of the indicated proteins in cerebellar extracts obtained from 5- and 10-week-old animals reveal that Atxn1 protein is induced in transgenic animals relative to wildtype littermates. Reflective of the decrease in Purkinje cells, Ronin transgene expression and Atxn1 induction were drastically reduced at 10 weeks of age compared to the same analysis at 5 weeks of age. (**C**) Quantification of endogenous Ronin protein levels relative to Gapdh levels shown in (B). Data are shown as box plots. Each sample is indicated by a circle; center lines represent medians. P-values were determined by two-tailed t-test. (**D**) RNA levels of Atxn1 in ES cells as detected by microarray analysis (Seifert et al., 2017); the result shows that Atxn1 transcript levels are higher in ES cells overexpressing wildtype *Ronin* when compared to control cells. As seen at the protein level, this increase is dependent on Hcf1. -Data represent mean ± SD. P-values were determined by two-tailed t-test. endo, endogenous; IP, immunoprecipitation; n.s., not significant; tg, transgene; wt, wildtype.

Disease Models & Mechanisms • Supplementary information

Table S1. RNA-seq of cerebellar extracts from 5-week-old animals
(A) RNA-seq of transgenic and wildtype control cerebella. Listed are all differentially expressed genes with an adjusted P-value of 0.1 or below. FC, fold change; padj, adjusted P-value; WT, wildtype control; TG, transgenic.
(B) Top 30 Gene Ontology (GO) categories enriched for Ronin target genes that are significantly upregulated 1.25-fold or more in cerebella expressing transgenic Ronin when compared to wildtype controls.
(C) DisGeNET categories containing genes that are downregulated 1.25-fold or more in cerebella expressing transgenic Ronin when compared to wildtype controls.

Click here to download Table S1

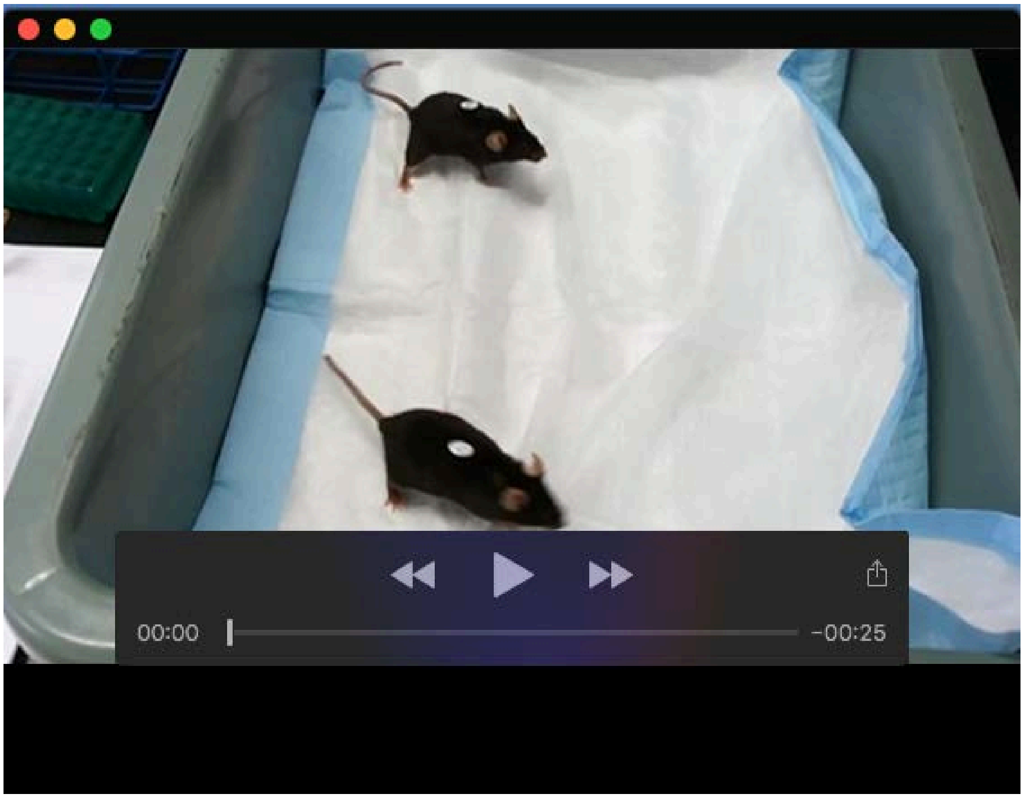

Movie 1. Recording of one wildtype (bottom) and one transgenic (top) animal walking in an enclosed area. The transgenic animal shows an ataxic gait, while the age- and gender-matched wildtype animal walks normally.
